# A Description of a Disease Called Charbon, That Prevails between the Sabine and Red Rivers, Communicated in a Letter to Dr. Sparks, from James Z. Ellison, M. D., of Opalousas

**Published:** 1830

**Authors:** James Z. Ellison


### Art. VI.—*A description of a disease called Charbon, that prevails between the Sabine and Red Rivers, communicated in a letter to* Dr. Sparks, from James Z. Ellison, M. D. of Opelousas.

Dear Sir,—

In my last letter I promised to give you some account of a disease prevalent in this section of country, called by the inhabitants *charbon.* Many physicians confound it with malignant carbuncle, but I am disposed to concur in the popular opinion that it arises from the bite of some insect and I am the more disposed to this belief, as Linneus has described an insect, under the name of Furia Infernalis, whose sting produces a disease very similar to the one under consideration. The marsh between these two rivers extends about 20 leagues from the Gulf of Mexico. It abounds with insects and the disease I believe is never met with more than 30 leagues from the Gulf.

The disease begins with a small pimple surrounded with an areola either red or livid, and a hardness in the cellular membrane under the integuments, with a troublesome itching, or a slight burning pain. The pimple in the centre shews a constant disposition to sphacelate, and forms a dark coloured eschar; the swelling extends and increases, and the pain becomes more acute and lancinating, and eventually is so excruciating, that it frequently destroys persons of robust constitutions in five or six hours. In the mean time the constitution is involved; great debility follows, the pulse small, weak, frequent and quick; the surface is cool, but the patient is tormented with a sense of internal heat and inextinguishable thirst; the countenance is sallow and expressive of the greatest anxiety and distress. As the disease advances, the prostration increases, the pulse becomes feebler and more frequent, the tongue dry and rough. The duration of the disease varies greatly. In mild cases the patient frequently recovers. In more violent ones, life is sometimes destroyed in a very short time—sometimes the fatal termination is protracted to the 5th or 6th day, or even longer.

This disease may be communicated in various ways. Besides the bite of the insect by which it is originally produced, it may arise from inoculation, or from eating the flesh of those animals which have died with it. Dogs are frequently said to die from the latter cause. A respectable farmer in this vicinity told that about 30 of his hogs, having eaten a small portion (not more than ten pounds) of a horse that had died of this disease, died in a very short time. It is currently reported that ravens will not eat the flesh of these animals. Whether this be so, or not, I have frequently remarked that although they are sufficiently numerous in this country to destroy the carcases of dead animals in thirty hours after they are cold, yet they often permit them to moulder untouched. Persons employed in skinning charbonic animals frequently catch the disorder. A physician in this vicinity who has had very extensive experience with the disease informed me, that a Spaniard having, by way of bravado, eaten a piece of the flesh of a beef that perished of this disease, died in a very short time, and the person who skinned it, having carried the skin for some distance on his back, caught the infection through a sore on his neck, and died in consequence. Whether it is ever caused, as the milk sickness is said to be, by eating the milk of animals affected with it, I have not the means of determining. The number of cases that occur from the sting of the insect is greater than those from infection. I have met with six cases of the former in man, and perhaps five or six times that number among the lower animals; and but one instance arising from the latter cause.

The treatment of this disease is principally local. Depleting remedies are not well borne, and stimulants are equally inadmissible, except in the last stages of the disease. All practitioners coincide in the propriety of destroying the eschar, although by different methods. Some preferring the knife, others the caustic. The latter is, I believe, in general the best practice. If the disease be large the Nitrate of silver, or the caustic potash, should be applied to the whole eschar, over this again we apply Aqua Ammonæ and a blister sufficiently large to cover the whole tumor. When the specific character of the disease is thus destroyed, the ulcer will generally heal under the common treatment. The constitutional treatment must be regulated by the stage of the disease, and the general character of the symptoms. Early in the disease before the constitution is much oppressed measures must be taken to reduce irritation and restore the secretions; keeping in view, in the use of remedial agents, the great debility that is likely to ensue. But I have already exceeded the limits prescribed to myself and must therefore leave the subject, with the hope that these hasty remarks may lead to investigation, and induce abler hands to bestow upon it, that attention which its importance calls for.

*Note by the Editor.*—As the writer of the above article has referred to a similar disease, supposed by Linneus to be produced by the Furia Infernalis, we will present to our readers the translation of an article, upon this subject, in the *Dictionaire Des Sciences Medicales.*

Furia Infernalis. The inhabitants of some parts of Sweden, and especially of Bothnia and Finland, are subject to a species of carbuncle which attacks, for the most part, the hands, the face, and other parts of the body that are left uncovered. The pain which accompanies this tumour is excruciating, and the consequences are sometimes so serious that they terminate by mortification and death. The celebrated naturalist Linneus was attacked by this malady in one of his journeys. A peasant informed him. that the disease was produced by a kind of worm, which was carried by the wind upon men or animals, penetrated their flesh, and could only be expelled by the suppuration which its presence excited. He shewed him also the worm which was the cause of his disease, dried, and about four lines long. Linneus, upon this information, admitted this insect as the cause of the sufferings he had experienced, and describing it under the name of *furia infernalis*, assigned it the following characters. Body linear, equal. fili, form, ciliated on each side with a single row of reflected prickles, pressed close to the body. The naturalists of Sweden at this day agree unanimously, that Linneus misled by the violence of the pain has too hastily assented to a popular prejudice; that the malady with which he was attacked was a species of pustule analogous to the carbuncle, which appears especially in autumn in the marshy parts of Sweden; that the pretended insect to which the people attribute the cause of this tumour is nothing but the apostume of the carbuncle, and that the animal presented to Linneus, was nothing more; or perhaps was the larve of an insect, which Linneus in its state of desiccation could not detect.

The respect which the Swedes very justly entertain for the memory of this illustrious naturalist, who has done so much honor to their country, has prevented them from directly contradicting the error into which he has fallen. But their naturalists generally admit, that the researches made to discover the *furia infernalis* have proved ineffectual; that the countrymen know it by its effects, but that no one affirms that he has seen it.

It appears that the species of tumour which has given rise to this error, although it sometimes terminates by gangrene, and is perhaps under some circumstances fatal, connot be classed among tumors essentially gangrenous, such as the anthrax, the malignant pustule of Burgundy, that of the Alps described by Mr Bayle, &c. for the application of simple emollient poultices is commonly sufficient to prevent the termination by gangrene.

